# Sexual and reproductive health risks amongst female adolescents who use amphetamine-type stimulants and sell sex: a qualitative inquiry in Yunnan, China

**DOI:** 10.1186/s12954-015-0065-y

**Published:** 2015-10-16

**Authors:** Xu-Dong Zhang, Angela Kelly-Hanku, Jia-Jia Chai, Jian Luo, Marleen Temmerman, Stanley Luchters

**Affiliations:** International Centre for Reproductive Health, Department of Obstetrics and Gynaecology, Ghent University, De Pintelaan 185, UZP114, 9000 Ghent, Belgium; Centre for International Health, Burnet Institute, 85 Commercial Road, Melbourne, Victoria 3004 Australia; International HIV Research Group, School of Public Health and Community Medicine, UNSW, Sydney, 2052 Australia; Sexual & Reproductive Health Unit, Institute of Medical Research, PO Box 60, Goroka, EHP Papua New Guinea; City College, Kunming University of Science and Technology, East Ring Road 50, 650051 Kunming, China; Department of Prevention and Education, Yunnan Institute for Drug Abuse, Xi Hu Lu 300, 650228 Kunming, China; Department of Epidemiology and Preventive Medicine, School of Public Health and Preventive Medicine, Monash University, Alfred Hospital, Commercial Rd, Melbourne, Victoria 3004 Australia

**Keywords:** Amphetamine-type stimulants, Female adolescents, Commercial sex, Sexual and reproductive health, Harm reduction, China

## Abstract

**Background:**

China, as other Southeast Asian countries, has witnessed an increased use in amphetamine-type stimulants (ATS) amongst urban youth. Amongst female adolescents who both sell sex and use ATS, risk behaviours are compounded resulting in even poorer health outcomes. However, limited knowledge exists on ATS use patterns and ATS-related risk behaviours, particularly in this context. This research aimed to improve the understanding of these issues amongst female adolescents who use ATS and sell sex, and to inform future programming.

**Method:**

This study utilised monthly focus group discussions (four in total) with the same study participants in Yunnan, China. From within a drug-treatment programme, female adolescents who reported both a history of drug use and selling sex were purposively enrolled in the study.

**Results:**

Participating adolescent females were aged 17–19 years and were all internal-migrants with low literacy. All reported polydrug use (mainly methamphetamine and heroin, whereas ecstasy and ketamine have been infrequently employed). Being less informed about risks of drug use and lack of sexual and reproductive health knowledge seemed to contribute to problematic drug use, rough and prolonged sexual intercourse, inconsistent condom use and ineffective contraceptive practice. For their income, participants largely relied on selling sex, which was frequently coupled with drug sharing services to clients. However, despite the practices, women did not self-identify as sex workers, and therefore did not think that existing intervention services targeting female sex workers were relevant to them. Moreover, criminalization and stigmatisation of drug use and selling sex impeded their access to care services.

**Conclusion:**

Current harm reduction and HIV/sexually transmitted infection (STI) prevention services are unlikely to address the demand of female adolescents engaged in drug use and commercial sex. Our findings highlight that a comprehensive and coordinated harm reduction and sexual and reproductive health response should be conducted involving these most vulnerable adolescents.

## Background

In the past two decades, there has been a significant increase in the availability and use of amphetamine-type stimulants (ATS) globally. Regions with the greatest increase include North America, Europe, Southeast Asia and Australia. Of an estimated 200 million people worldwide who used drugs in 2009 and 2010, around 35 million used ATS. This is more than those reported to have used cocaine (13 million) and heroin (16 million) combined [1]. ATS use in Southeast Asia has been particularly high with more than 60 % of global ATS use taking place in the region [2]. ATS use can cause a range of immediate and long-term health consequences to individuals through increasing high-risk sexual behaviours or other risky behaviours, such as unsafe injecting, or polydrug abuse. The health consequences have been well documented, including increased acquisition of HIV and other sexually transmitted infections (STIs), hepatitis B and C, tuberculosis and mental health problems [1, 3–6].

The People’s Republic of China has a long and evolving history of drug use [7, 8]. Historically, opiates were the primary used drugs, but as China began to open up its previously closed borders in the past 10 years, access to and demand for synthetic drugs have expanded exponentially [8, 9]. While use of heroin has decreased amongst urban Chinese youth [6, 10], the use of ATS, in particular, has become popular and has been mostly consumed in entertainment venues [6, 10]. Many entertainment venues provide easy access to illicit drugs in big cities of China [11, 12]. Since 2010, police reported arresting more people using crystal methamphetamine than those using heroin in 16 of China’s 31 provinces. Throughout China, the drug market has been, and continues to be, a significant challenge to both national and provincial law-enforcement agencies. China has taken a punitive and hard-line strategy towards drug use and drug trafficking. This is best exemplified in its ‘zero-tolerance’ law-enforcement approach on drug control. Based on the *Narcotic Control Law of the People’s Republic of China* (2008), the Chinese government has intensified its efforts to curb drug supply by strengthening the National Narcotics Control Commission and local governments [7, 13]. A hard-line approach against drug users has been adopted by the law-enforcement sector resulting in compulsory detoxification treatment, physical exercise and manual labour at mandatory detention centres [6, 7, 14–16]. Moreover, the commission has also implemented a specific action plan to strengthen the surveillance and reporting system for more effective screening, registration and monitoring of drug users nationwide. As a result of the rapidly expanding ATS market and intensified law-enforcement activities, the total figures for methamphetamine seized and the number of arrested and registered ATS users are continuously increasing [13]. According to police surveillance systems, by the end of 2012, China reported nearly 2.1 million registered drug users [13]. Of these, over a third (38 %) were identified as polydrug users (mainly methamphetamine and ketamine) [13]. Amongst all registered drug users in China, the proportion who use ATS is increasing. Within the 2-year period from 2008 to 2010, the proportion of registered ATS drug users more than doubled from 9 to 19 % [11]. Of particular concern is also the surge of young people using drugs, ATS in particular. In 2011, slightly less than one in five (18 %) were below the age of 20 years [13]. Also, of all registered illicit drug users in China in 2011, almost three quarters (73 %) were between the ages 16 and 25 years [8]. As these data only reflects the situation of those ATS users who have engaged in drug-related criminal activities and who have been caught, it is hard to assess the broader situation of ATS use amongst young people, nor are we able to ascertain more official information about sub-populations of ATS users, such as young female sex workers (FSWs).

Seizures of methamphetamine pills in China have also shown a considerable increase during the past few years, with around half of seizures having occurred in Yunnan, a southwestern province of mainland China, which borders with Myanmar, Vietnam and Laos [8, 11]. Traditionally famous for its ethnical diversity, Yunnan is now notorious as the epicentre of China’s drug trade, as it is geographically positioned along the major drug trafficking routes of the infamous ‘Golden Triangle’ [14]. For this reason, we have undertaken our study in this setting.

Beyond the legal sector, the health consequences of drug abuse also pose a notable concern for the public health sector. By the end of 2011, approximately 780,000 people were reported to live with HIV in China, of whom 28 % were reported to be exposed through injecting drug use and 64 % through sexual contact, including both heterosexual and homosexual modes [15]. To confront the dual epidemics of injecting drug use and HIV, especially where they coalesce, China initiated a pilot harm reduction programme just over 10 years ago (2004), albeit largely confined to methadone maintenance treatment. By the end of 2011, China’s methadone maintenance treatment programme has expanded to include more than 738 clinics across 28 provinces and has served more than 344,000 drug users [14–17]. However, methadone maintenance treatment is designed only to treat and support opioid-type drug users, not ATS. Since 2004, multilateral needle and syringe programmes have been piloted in selected parts of the country, but the withdrawal of international development investments in 2012 has resulted in a significant reduction both in size and number of the programmes, exacerbating already low overall coverage [18]. As with neighbouring countries, the harm reduction approach currently used in China fails to address the harmful ATS abuse and related healthcare needs for young people [2, 6, 7].

There is no definitive profile of a ‘typical’ ATS user, but there is growing evidence suggesting that those involved in the entertainment industry, such as FSW and young people, are especially vulnerable to ATS use [1, 4, 5, 19, 20]. Numerous studies have now identified a strong interrelationship between sex work and substance use [4, 6, 12, 21–26]. Sex workers may use substances to alleviate a range of problems from controlling weight, enhancing work performance, coping with the accelerated pace of life (irregular or late hours) to increasing sexual excitement and energy for sexual activities and easing inhibitions for multiple or disagreeable clients [3, 4, 27, 28]. Also, some women are motivated to sell sex for generating money to buy drugs, which is most likely associated with drug dependence, in this context, and could be viewed as a subgroup of drug users [27]. Compared to other drug use, understanding of ATS is limited. The two studies that do document ATS use amongst FSWs in Yunnan province identified that 16 % of participants reported having ever used drugs. Of these, about 7–9 % were injecting drug users (IDUs) [21, 26], similar to other southern Chinese cities [29]. There is little known about the prevalence of ATS use amongst FSWs in China. One study in Guangdong province identified that ecstasy prevalence was about 8 % amongst FSWs [30]. Evidence from the region, including Vietnam, Cambodia and China indicates that FSW who use ATS face a number of related poor sexual health outcomes including increased risk of HIV acquisition as well as other STIs [2–4, 27, 28, 31].

Due to social norms, cultural taboos and political sensitivities towards adolescents who are involved in sex work and drug use, this vulnerable population has often been overlooked within China’s harm-reduction policies and programmatic responses to illicit drugs and reproductive healthcare [1, 6, 18, 19, 27, 32], and has rarely been the focus of research. Therefore, there is a lack of understanding regarding patterns of ATS use, its relation with sexual risk taking and sexual and reproductive health challenges faced by female adolescents who sell sex and use drugs. This knowledge gap significantly impedes programmatic and policy responses which could mitigate potential harm and promote the health rights for these female adolescents.

A small qualitative study was undertaken amongst female adolescents aged 19 years or younger who were undergoing compulsory detox for drug use and who also reported a history of selling or exchanging sex. This research aimed to better understand the culture of ATS use in order to inform evidence-based programming for female adolescents with a multitude of risk practices.

## Method

### Study setting

There are 45.7 million people (2009) living in Yunnan Province with 6.4 million in Kunming, the largest economic and cultural centre and the provincial capital. Despite only making up 3.5 % of the national population, Yunnan Province accounts for 22 % of new HIV cases in the country [15, 33] with HIV prevalence rates concentrated amongst IDUs (28.4 %) and FSWs (2 %) [34].

It is estimated that there are more than 80,000 people in Yunnan Province who inject heroin [35]; however, there are no official numbers which estimate or report the number of ATS or polydrug users. As of the end of 2009, Yunnan had over 70 compulsory drug detoxification centres administrated by the Public security. By 2010, the Kunming Compulsory Drug Detoxification Center (KCDDC) was the largest centre in all of China with an estimated 3000–5000 male and female ‘inmates’ [35–38]. A person who used drugs and is arrested is usually detained and ordered to undergo compulsory urine drug testing at a police station where they are then transferred to methadone maintenance therapy clinics, supervised at community-based detoxification centres for at most 3 years or incarcerated in mandatory detoxification centres for at most 2 years [7, 39], for example, the KCDDC. Due to the high relapse rates following release from these compulsory drug detoxification centres, the Kunming Rehabilitation Center is managed by the Department of Public Security and serves as a transition place to prevent the relapse for released ex-drug users from the KCDDC. Under the legislation, after their release from KCDDC, ex-drug users can select contracting with an authorised community-based therapeutic facility or Kunming Rehabilitation Center. Individuals who stay in the Kunming Rehabilitation Center are subjected to strict rules, are not permitted to leave freely during their stay and undergo random drug testing, behaviour change programmes and physical exercise. As its programme and regulations suggest, the Kunming Rehabilitation Center is considered an incarceration site. During our research, the observed monthly number of individuals in the centre was around 50 with equal ratios of men and women.

Though it is conservatively estimated that there are more than 10,000 active FSWs in the urban areas of Kunming, all forms of commercial sex remain illegal throughout the country [40–44]. Based on their observation and work with FSW, a local peer-led organisation of sex workers recently estimated that at least 30 % of the women they reach in their programmes were adolescents.

### Study design and data collection

During the research period, it was logistically not feasible to recruit female adolescents who are selling sex and using drugs through existing FSWs’ or IDUs’ support groups or programmes. Instead, our study participants were recruited at Kunming Rehabilitation Center from amongst 20 adolescent female individuals. Participants were eligible to participate if they were aged 19 years or younger, self-reporting a history of ATS use and selling or exchanging sex before entering KCDDC and were willing and able to participate and provide informed consent for this research. Moreover, women were excluded if they had an obvious mental or other medical illness that would impede participation (as judged by the medical department of Kunming Rehabilitation Center).

Between March and July 2011, a qualitative inquiry was conducted using repeated focus group discussions (four sessions in total) with the same participants, anticipating increased yield on these highly sensitive topics with repeated exposures. Repeated group discussions were found to be conducive for gradually developing rapport and trust between the participants and researcher(s) with participants disclosing more as the groups went on. This indicates a level of comfort had been reached with the female adolescents in discussing sensitive topics and illegal activities. Moreover, this method reduced recall bias through comparing or re-discussing relevant topics in different sessions, therefore enhancing validity of data in comparison to one-on-one interviews or once-off focus group discussions. In addition, this approach could stimulate and elicit new important topics further refining the initial interview guide for subsequent interview sessions before the fieldwork had been completed.

The group discussions were facilitated by a semi-structured interview guide which encouraged participants discuss the relevant events and emotions, behaviours and knowledge. Core topics discussed included the following: participants’ socio-demographic background, perspectives and practices of drug use; sexual and contraceptive practices; sexual health; health-seeking behaviours; and self perception and future aspirations. Discussions started with broad personal topics and then evolved to more sensitive topics in a supportive and non-judgemental way.

A typical focus group discussion lasted around 1.5 h. All discussions were moderated by the lead author together with one of two trained note-takers. All focus groups were digitally audio-recorded, transcribed into Chinese Mandarin and proofread by note-takers. Chinese transcripts were read through multiple times, summarised and then thematically coded. Twelve primary themes and 26 sub-themes were identified. Coding was cross-checked by other authors fluent in Chinese.

### Ethical consideration

Ethics approval was obtained by the research ethics committee of the Kunming Public Health Bureau, Yunnan Province, China (No. KM-FSW-10-06).

A brief introduction and ethical materials about the study were provided to Kunming Rehabilitation Center for review and permission. A pre-discussion was held between the research team and Kunming Rehabilitation Center staff on how to meet administrative requirements while protecting participants’ confidentiality; the procedure of recruitment and interview were also discussed. Kunming Rehabilitation Center staff contacted all eligible inmates and asked if they were willing to participate in this study voluntarily.

The venue, schedule, length and form of sessions were determined in consultation with six candidates. Written informed consent of participants was obtained before each session; there were three candidates who were under the age of 18. Given they were living apart from their parents for years and without current connection with family, the written informed consent was obtained from a trusted adult selected by the participant in line with WHO’s ethical considerations in conducting research amongst adolescents in developing countries [45], as well as the acknowledgement on the evolving capacities of children outlined in the Convention on the Rights of the Child [46].

Confidentiality and privacy were of most concern in this study. Both oral and paper statements were given to each participant to assure the confidentiality of collected materials and the understanding of the study. All questions asked by the moderator were conveyed in plain local language in relation to their age and life circumstances. Participants were clearly informed about the voluntary nature of their involvement and their right not to answer some questions as well as to withdraw at any time without jeopardising their treatment and care at the Centre. All discussions were conducted in a private meeting room within the Kunming Rehabilitation Center, with refreshments, nearby toilets, and comfortable seating. None of the staff from Kunming Rehabilitation Center was present during the discussions, nor did the research team share conversations and issues with them.

As per rules from the Kunming Rehabilitation Center, participants were not allowed to receive money for their involvement in the study so at the end of each session, participants received a toiletry bag with personal hygiene products equivalent to the value of RMB30 (US$5) in appreciation of their time and participation. Furthermore, participants received HIV/STI counselling and family planning/contraceptive advice and information as requested, as well as an information sheet of local support organisations for female sex worker and IDUs.

## Results

### Characteristics of participants

Six female adolescents were eligible and consented to participate in the study. The duration women resided in the Kunming Rehabilitation Center varied from 6 months to a year. All self-reported behaviours regarding drug use and sex trade were prior to the detention in KCDDC. All six were Han rural-urban migrants aged 17 to 19 years, and most of them had not completed middle school (Table 1). None of the adolescents reported to have a connection with family when they were arrested for fear of disclosing their life situation with selling sex and drug use. Prior to arrest, their main source of income was from selling sex and/or shared drug use services to clients. Another reported source of income was living of the earnings of introducing other girls to clients for either or both sex or shared drug use. At varying times in their lives, in the absence of income from above sources, participants reported having been involved in other criminal activities, such as selling drugs, stealing and extortion to earn money. The reported monthly income varied from US$1200 to US$4800.^1^

**Table 1:** Key characteristics amongst adolescent female sex workers who ever used ATS

Key characteristics	n/N
Age	
17 years	3/6
18 years	2/6
19 years	1/6
Education	
Completed primary school	1/6
Not completed middle school	4/6
Completed middle school	1/6
Residential status	
Rural areas in Kunming	3/6
Other rural areas in Yunnan	3/6
Current connection with family	0/6
Ethnicity	
Han	6/6
Social insurance (medical care, social relief)	0/6
Type of drug used	
Methamphetamine pill	6/6
Crystal methamphetamine	2/6
Ecstasy	6/6
Ketamine power	3/6
Heroin	6/6
Drug use history	
Less than 1 year	2/6
1–2 years	2/6
More than 2 years	2/6

### Use of amphetamine and other drugs

#### Types, effects and perceptions of drugs used

All participants were polydrug users, and the most frequently used ATS was methamphetamine. Known locally as ‘xiao-ma’ (‘little horse’), the pill containing methamphetamine was usually opened and the content deposited onto a piece of foil and smoked. The second most common ATS used was crystal methamphetamine (‘ice’), known locally as ‘bing or bing-du’. The women referred to high purity crystal methamphetamine as ‘shui-guo-bing’ or ‘bai-bing’ (‘fruity ice’, more transparent ice with a little sweet taste) and the impure as ‘hua-xue-bing’ or ‘huang bing’ (‘chemically synthesised ice’ with a light yellow colour). A plastic drink bottle with two straws was often used to smoke ice after it had become gaseous and had been filtrated through water. In colloquial Chinese, this is called ‘liu-bing’ (‘feeling like skating’).

Initiation of ATS use was often from peers who introduced them to all-night ‘horse parties’ where methamphetamine pills and crystal methamphetamine were provided, usually in abundance. The most common reasons for experimenting with ATS included expecting euphoria and less sexual inhibitions as well as increased excitement, energy and sexual desire. Typically, the female adolescents reported that they would smoke anywhere between four and ten methamphetamine pills at any one time. Sometimes, they would use methamphetamine pills and crystal methamphetamine simultaneously. The frequency with which they took ATS was largely dependent upon two issues: having the financial means to afford ATS and; if a sexual client desired for her to take it with him. In the latter circumstance, the client would purchase the drugs for the girl’s consumption.

The female adolescents described in detail the short-term effects that methamphetamine use had on their physical and mental health. Typically, these effects included increased bodily temperature, excessive sweating, dizziness, insomnia and hallucinations. These effects of methamphetamine experienced were different depending on the purity of the drug consumed. Women reported that the low-grade methamphetamine was seen as less potent and posing quite uncomfortable symptoms including itchiness, redness of the body and numbness of the throat. In contrast, high purity methamphetamine was reported as making them feel euphoric and invigorated, with less or without uncomfortable symptoms.

Apart from using ATS, all female adolescents reported a history of frequent heroin use. Usually, heroin was introduced by peers or a boyfriend in order to combat the euphoric ‘high’ from ATS, particularly when they wanted the ATS side-effects to end, as expressed by one participant:

I used heroin after horse parties [methamphetamine parties] or after sharing little horse [methamphetamine] with clients to help me sleep well.

Despite access to heroin for over 2 years in some cases, there were no reports of injecting drug use nor did any of the participants report an overdose. The reasons for not injecting heroin included feeling troubled to prepare a venous injection, and fear of needles or pain. Participants did, however, report two cases of their closest girlfriends injecting heroin, in which one collapsed and another died. This experience had an enormous impact:

…[I was] thoroughly scared and, don’t know how to help her.

Ecstasy (in colloquial Chinese, ‘yao-tou-wan’) was less often reported, as well as ketamine (‘K-fen’, in pure powder form) were occasionally used by all participants when they hung out:

…in some night entertainment venues for fun with other club-hoppers.

The participants contrasted the addictive and harmful nature of heroin with ATS, whereby heroin was interpreted as a ‘hard drug’ and therefore more serious in its effects and its addictive nature. In contrast, they perceived ATS as largely harm free, fashionable, fancy and cool, sometimes described as drugs that ‘belong to the new generation like us’.

#### Costs and venue of drug use

The cost of different drugs varied greatly. Methamphetamine costed between RMB 40 and 50 (around US$6–8) per tablet. The cheapest low-grade methamphetamine was available for around RMB 25 and 40 (around US$4–6) per tablet. Cost of drugs was not always the burden of an individual female adolescent. Purchasing drugs was often seen as the responsibility of the girls’ boyfriends as one participant shared:

We always share the cost of drugs with our girlfriends; but with boyfriends, usually they pay for this [drug cost], if he didn’t want to pay, I shall refuse to use drugs with him.

Similarly, drug-taking peers or clients of sex services often supplied the participants with drugs:

You know, sometimes my friends will bring me some ‘new arrivals’ or ‘finest goods’. We could try some and buy some more from them; but actually, many clients usually bring drugs to share. We don’t need to pay.

Few participants in the study reported that they had taken crystal methamphetamine, methamphetamine tablets or heroin in their home; but most sought out other venues including hotel rooms rented out by the hour and small road-side guesthouses to take drugs. These venues were chosen for their secrecy, of being able to avoid the gaze of law-enforcement officials and prying neighbours. Other important venues for drug-taking included entertainment-settings, as one girl described and was reinforced by others:

There are some private suites, each suite with a lobby, and a separate backroom with a toilet typically, the lobby for dancing and karaoke, well, also for ‘liu-bing’ [smoking methamphetamine pills] together; the small backroom for ‘fast food’ [a quick sexual intercourse for 15-20 minutes].

For ecstasy-type tablets, it was most often consumed in nightclubs, dance clubs or rave scenes as a participant recalled:

…these pills are colourful things with very cute and attractive patterns, ah, exuding a pleasant aroma. You could buy it either from drug dealers peddling around in these club scenes, or from your familiar peers; well, the first way is unusual during police crackdown.

### Sexual and reproductive risks related to ATS use

There was an identified relationship between methamphetamine use and the participants’ sexual behaviour. All reported that using methamphetamine or crystal methamphetamine meant they experienced heightened sexual libido and increased sexual energy. Sexual intercourse was prolonged, and multiple sex acts could occur in one encounter/night.

…we think having sex is a good relaxant to [complement] the effects of ‘little horse’ or ‘ice’. You know, we call this way as ‘jie-ma’ or ‘san-bing’ [soften the euphoric high coming from methamphetamine or crystal methamphetamine]. I feel my body is very hot with intensive desire for it [sex] after I take ice or little horse. Well, they [sexual partners] say my behaviour is very crazy and impulsive. We often take drugs together so we could have longer sex, for example, two to four hours or usually over five sex acts during the night.

Obviously, there were adverse health consequences related to the above sexual activities after methamphetamine use as evident in the extracts below:

I had sexual excitement but could not easily get a ‘high’ feeling [orgasm], so we want the long-lasting sexual intercourse, oh, sometimes, I feel burning pain on my labia after drug effects.

Myself and some of my girlfriends, we have had experience of abrasions and tearing around my ‘below areas’ [vulva or perineum] with prolonged sexual intercourse.

Another problem regarding their sexual practices was the inconsistent condom use with sex clients and the rare condom use with boyfriends (the majority of whom were also drug users). Condoms were mainly perceived as a method for the prevention of unwanted pregnancy. Interestingly, a number of the participants described that male drug users have lower fertility, so traditional methods (i.e., douching, herbal mixtures, withdrawal or rhythm) were perceived to be ‘enough’ to prevent conception in such instances. The reported level of contraceptive practice was unreliable. Participants reported to not use the oral pill because of the concerns about reduced future fertility, the resulting ‘acne’ and ‘ugly chloasma’ as heard from other peers, as well as the potential difficulties in compliance. None of the female adolescents reported having heard of, or using, an intrauterine device or implant.

Unwanted pregnancies and induced abortions were reported by three participants. Nearly four out of five of their female peers^2^ had had at least one unwanted pregnancy which often ended in an induced abortion, though a few gave birth. One participant described a girlfriend even experiencing 12 induced abortions.

None of the young women had asked if any of their boyfriends had tested positive to HIV or had been diagnosed with other STIs. In fact, these female adolescents explained their failure to enquire about these health matters because the men they dated ‘looked healthy’ and therefore not at risk of being positive to any such infection.

### Self perception

Female adolescents discussed how they saw themselves within the Chinese cultural context in which considerable stigma surrounds FSWs, even in drug users’ communities. Describing themselves as ‘horse girls’ , those who provide combined services of having sex and sharing drugs positioned themselves as superior to those women who only sold sex. Horse girls were more likely to identify themselves as drug users rather than sex workers. Compared to these who just sold sex, horse girls perceived their ‘professional’ skills, their specific client networks, and their high income and financial independence as more control and flexibility over their client selection, thus contributing to their superior status:

This [sold sex] was for fun, and I mean I don’t think of it as my occupation for making a living; I think we are different from those professional FSWs who have to provide low-price sex with clients, no matter if they like or dislike. They seem to have ‘no taste’ , and are ‘humble’ without choice, it is a pity. I can decide and select the clients I like, so I don’t think it could be called ‘work’.

I don’t think these [FSWs] are our kind of people, they [FSWs] can only sell their ‘flesh’ [bodies] …and they are always held in contempt in our circles (drug users).

I will lose my face in front of people who know me if I bear this kind of disrepute of being a ‘Ji’ [prostitute in colloquial Chinese]. In this situation, my family will be in complete disgrace and I have no chance to marry a good guy.

Well, we all know, if you meet clients who bring “ice” or ‘little horse’ along, or already used them, you have to take them [ice or little horse] too; if not, you could not bear the sexual intercourse for hours. They [clients] call us ‘ma-mei’ [horse girls], …well, only girls like us can handle this, I mean our skill in providing clients with drug-related services is a special profession compared with providing sex services alone, we called this business as ‘pei-hi’ [accompany with ‘high’].

In some entertainment places, girls like us [horse girls] have our own lounge to wait for special clients [drug using], other ordinary FSWs [without drug related services] are not allowed to stay in the room, even if we all were working at the same entertainment place. The managers or owners try to keep us away from strangers, or not so trusting persons to protect us, you know, they don’t want to get trouble from police.

### Healthcare seeking behaviour

With the fear to be exposed for their illegal behaviours of both drug use and selling sex, safety was the number one priority amongst these female adolescents. Participants instinctively avoided contact with government-led health services. Furthermore, judgemental attitudes of health providers, inconvenience and lack of medical insurance were reported to result in poor uptake of public healthcare services. In this regard, female adolescents described:

Actually, I never heard of any place that can provide free HIV counselling and testing services, nor sexual transmitted infections testing and treatment. Well, I heard of something about methadone program from my friends. I don’t know when health workers will come to our working venue because I have taken few chances to meet them for ‘security’ reasons. Only once, I heard they were distributing condoms to FSWs, so I quickly went downstairs to get some free condoms from a woman [outreach worker] without talking to her.

We are familiar [with small private clinics], we are comfortable with the doctors’ attitude and patients, well sometimes we can even bargain against the cost. Unlike those big hospitals [public], too many patients, too many procedures with too many questions about my occupation, my address, my contact number, and my marital status or partners. I would not bear such eyes and tones. And, we have no medical insurance; I pay all the cost, so why do I have to go to these big hospitals?

When asked how they deal with these negative consequences, one girl reported:

It is not necessary to suffer from this [seeking health services in public hospitals], and I don’t want to tell the doctor about my experience about the injuries. I feel unsafe to do so…and if very painful [injuries], I would go to the pharmacy to buy some jie-er-yin [a kind of over-the-counter vaginal douches for bacterial vaginosis treatment]; if mild, I would not care.

All participants reported that they received compulsory testing for HIV while entering KCDDC like other inmates. However, the test results had not been communicated to the participants, and they were left not knowing their HIV status.

Self-detoxification experience was commonly reported by all participants, as many as eight times. The main reasons for detoxifying this way included difficulty in finding any reliable facilities (government-funded or private) that provide ATS-specific treatment and information, confidentiality or safety concerns about these services.

## Discussion

### Summary of findings

Our findings show that these female adolescents’ vulnerability to drug use and health risks is rooted in poor social support and structural factors.

#### Stigma, criminalization and self-identification

Our study identified that the dual criminalization of sex work and drug use have driven these female adolescents to be a ‘hidden’ group. This is not only reflected in disconnection with their families and the high mobility of drug use venues, but also in the low uptake of public healthcare services for drug treatment, reproductive tract infections, abortion and contraception. Moreover, the discriminatory policy in service provision of family planning, the judgemental attitudes and inadequate counselling skills of public health providers, as well as the lack of confidentiality and health insurance all contribute to these adolescents not accessing public health services.

The horse girls in our study did not identify themselves as sex workers despite the sexual services they provide. Participants’ self-identification as drug users rather than sex workers also reflects the female sex workers bear even more stigma in the Chinese context. This is also an important dynamic in the perception of existing public health services targeting FSWs, which were perceived to be irrelevant for them.

Smoking was the predominant method for taking ATS in this group. Similar to a previous study [5, 18], they rarely used harm reduction services, largely because the existing harm reduction services have been designed targeting opioid users and injecting drug users. Obviously, mainstream interventions which solely either focus on IDUs or focus on adult FSWs failed to reach this population. This situation further fuels their marginalization and enhances their vulnerabilities to a myriad of poor sexual health outcomes including HIV, other STIs and unwanted pregnancies.

#### Socio-economical pressure

All female adolescents in our study were rural-urban migrants with relatively low education level, and were living apart from family and out of formal schooling. Therefore, these adolescents did not enjoy the presumptively protective environment from family and school. As the newcomers in big cities, they were facing life-changing, economic hardship and negative peer influence. All participants were self-supporting, and the sex services coupled with shared drug use constituted the largest contribution to their income. In contrast to wages earned from potential low-skilled jobs (which they could likely access in urban areas) or sex work without drug involvement, their income was considerably higher and provided more economic freedom and independence for these female adolescents. It might therefore be difficult for these adolescents to separate drug use and sex trade from their livelihood. In efforts to address such challenges, it is not simply a case of solely relying on the law-enforcement approach and criminal justice system without further family and social support.

#### Scant knowledge on sexual health and drugs

Our study showed these female adolescents were involved in a number of activities that would be deemed risk behaviours, yet this is not how they described it. Their ATS use, and in fact their polydrug use, were regarded as largely harmless. Also, the limited apparent familiarity with long-acting reversible contraceptives and their reliance on inconsistent condom use for pregnancy prevention meant that unintended pregnancies seemed rather common. The belief that drug-using partners were less likely to be fertile further led to decisions of unprotected sex. In China, the government-funded family planning services are only accessible to married couples [47–50]. The traditional social-cultural taboos deter the provision of sexual and reproductive health (SRH) education and drug knowledge at schools and at home. As a result, adolescents are unlikely to be prepared to protect themselves from adverse health consequences as they engage in sexual relationships or drugs experimenting.

Alongside these identified structural factors, the evidence of adverse health consequences related to ATS use emerged from this study. Consistent with previous studies [51, 52], our findings show the amplifying effects of methamphetamines on sexual libido and desire. Moreover, the reduced sensitivity of the genital area and lengthened duration until orgasm both facilitated longer and rougher sexual episodes. As a result, the symptoms reported of bruising and tearing of the genital areas could further increase risk of HIV/STIs acquisition. This situation is coupled with the fact that this group reluctantly accessed existing public healthcare services, which suggests that these female adolescents are at great health risks.

Our findings showed that these female adolescents were less likely to engage in injecting drug use. However, some reported cases amongst their close peers involved overdose, one of which was lethal. Available data suggest the crystalline form of methamphetamine poses a high dependence potential. In some Southeast Asian countries, ‘smoking’ crystal methamphetamine users are at the increased risk of progressing to other more harmful routes, for example, transition to injecting [52]. Our study suggests that preventing the uptake of injecting drugs should be a primary aim of harm reduction in this group of adolescents.

Moreover, findings also suggest taking into account the interrelation between these female adolescents and their sexual partners (boyfriends or clients) regarding harm reduction. Consistent with other research amongst adolescents [53–58], our study shows female adolescents relied on emotional feeling or observation to judge the health status of their sexual partners. This, in combination with the fact that a number of their sexual partners were drug users, shows potential risk to HIV/STIs acquisition. When taking an insight on drug use behaviour of horse girls, their sexual partners play an important role in drug supply. Sexual partners often supplied drugs for free, which meant these horse girls loose control of dose, quality, pattern of use, and the consuming venue. Each of these cases could not only inhibit their ability to negotiate safe sex, but also expose them to the elevating risks of overdose and sexual and physical abuse.

### Implications

There are several key elements for tailored programmes to make a difference for this population.

#### Changes of laws and policies

It is important to review or reform legal and health policies to facilitate the effective responses and promote human rights for women engaged in selling sex and using drugs, particularly for adolescent females. First and foremost, global evidence shows that a punitive approach to drug use is not effective in addressing the complex social and health factors associated with drug use, while negatively affecting public health responses [28, 44, 45, 51]. Harm reduction, a public health and rights-based approach to mitigate long-term harm should supersede a punitive approach, which largely focuses on supply and demand reduction [1, 5, 9, 52]. Discriminatory health policies present significant barriers to this population. Removing the restriction on unmarried young people’s access to government-subsidised family planning services in China is a key priority to improve the SRH and rights of female adolescents, including those engaged in the sex trade and drug use.

Beyond these structural barriers, historical tensions between SRH promotion and moral repercussions must be overcome. It is essential to invest both in sexual and drug education based in schools and healthcare settings to increase adolescent girls’ knowledge and protective skills and build-up their decision-making power.

#### Create new intervention platforms to reach the horse girls population

Existing HIV/STI interventions and harm reduction services should be reoriented to reinforce each other, hence improving accessibility and acceptability of health services for these female adolescents. As early as possible, an integrated programme should be conducted at a variety of HIV/STIs and harm reduction service settings. However, these programmes are not enough to reach out to this population as most horse girls did not regard themselves as FSWs or IDUs. Innovated measures to reach these most-at-risk adolescents effectively need to be developed, for example recruiting and training young peer educators/outreach workers to reach adolescent FSWs in their workplaces or living places; re-orientating drop-in centres operated by local NGOs to create safe places where the peer education and support, primary healthcare, adequate information and counselling and referral services are accessible; and providing youth-friendly healthcare services at community-based clinics or health centres in areas where adolescent FSWs connect or congregate. Given the nature of adolescent psychological and physical development, age-appropriate and non-judgemental information, counselling and services of gynaecology, contraception is essential.

#### Community empowerment

Government-led health programmes in China continue to act as the primary HIV prevention service provider [40]. Nevertheless, institutionalised discrimination and criminalization restrict this population’s access to government-led services. While the community-based and peer-led programmes are proven as an effective strategy to address key population’s unique health needs in many countries, including China [40, 52, 59–63], these were not available for our target population. Encouragingly, the impact of community-led FSWs/IDUs interventions in China suggests that, even under legislative and regulatory constraints, community-based HIV/STI prevention and harm reduction programmes have been successful in community mobilisation, drop-in centres operation, peer education and outreach, as well as bringing about a decrease in risk behaviours [62, 63]. Strengthening representation and involvement of the target population in policy and programme formulation can play an important role of community empowerment as shown in several low-income and middle-income countries [64]. Programme initiatives should ensure that these adolescent horse girls are involved in development, implementation and evaluation in accordance with their best interests to acquire skills, rights and empowerment.

### Limitations

These findings need to be interpreted in light of certain limitations. First, the study was limited to Kunming and participants were recruited from a correctional and incarceration setting. Caution is therefore needed in generalising the findings to other groups of female adolescents who only use drugs or sell sex, or without contact with these incarceration settings, or those in other regions. To our knowledge, this study is the first attempt to understand the culture of ATS use and related health risks in female adolescents involved in the sex trade. Given its exploratory nature and the challenging environment, findings provide interesting perspectives in spite of these limitations.

### Further research

Due to the increase in the number of young people or adolescents using ATS, further research should consider a qualitative or mixed method by involving more female adolescents with overlapping behaviours of selling sex and using drugs. Moreover, research on targeted intervention is urgently needed on how to effectively reach and provide these adolescents with health and social support services through community-led efforts, and how they can be more empowered to enable them protect themselves from negative physical, social and mental consequences. A better understanding is also needed to obtain details on context and content of the interaction with sexual partners or its influences behind their sexual and drug use behaviours to offer reliable explanations and implications for programming.

## Conclusion

Female adolescents involved in drug use and commercial sex suffer from dual stigma and criminalization, and remain overlooked and uninformed by conventional HIV/STI and harm-reduction programmes. Our findings confirm that structural changes are needed to remove barriers from accessing healthcare services and safeguard this population’s sexual and reproductive health and rights. When attempting to reduce the harms associated with drug use and sexual risk behaviours in this population, peer networks and community-based organisations are key to a successful intervention, and should be invested in. Our findings suggest that as early as possible, community-led and multi-sector-coordinated tailored harm-reduction programmes that address their unique circumstances, vulnerabilities and health are urgently needed to substantially mitigate them from adverse health and social consequences.

## Abbreviations

ATS: amphetamine-type stimulants
FSWs: female sex workers
IDUs: injecting drug users
KCDDC: Kunming Compulsory Drug Detoxification Center
SRH: sexual and reproductive health
STIs: sexually transmitted infections

## Footnotes

1 In 2011, the average monthly wage was RMB 3470 (US$580) in Kunming (source: Yunnan Bureau of Statistics).

2 This figure came from each participant reporting having roughly 10 closed female friends, and about ‘seven to eight’ or ‘majority’ of those friends had had one unwanted pregnancy.
